# Is there much variation in variation? Revisiting statistics of small area variation in health services research

**DOI:** 10.1186/1472-6963-9-60

**Published:** 2009-04-02

**Authors:** Berta Ibáñez, Julián Librero, Enrique Bernal-Delgado, Salvador Peiró, Beatriz González López-Valcarcel, Natalia Martínez, Felipe Aizpuru

**Affiliations:** 1 Fundación Vasca de Innovación e Investigación Sanitarias (BIOEF), Bilbao, Spain; 2 CIBER Epidemiología y Salud Pública (CIBERESP), Spain; 3 Instituto Aragonés de Ciencias de la Salud (IACS), Zaragoza, Spain; 4 Centro Superior de Investigaciones en Salud Pública (CSISP), Conselleria de Sanitat, Valencia, Spain; 5 Departamento de Métodos Cuantitativos, Universidad de Las Palmas de Gran Canaria, Las Palmas, Spain; 6 Unidad de Investigación, Hospital Txagorritxu, Vitoria, Spain

## Abstract

**Background:**

The importance of Small Area Variation Analysis for policy-making contrasts with the scarcity of work on the validity of the statistics used in these studies. Our study aims at 1) determining whether variation in utilization rates between health areas is higher than would be expected by chance, 2) estimating the statistical power of the variation statistics; and 3) evaluating the ability of different statistics to compare the variability among different procedures regardless of their rates.

**Methods:**

Parametric bootstrap techniques were used to derive the empirical distribution for each statistic under the hypothesis of homogeneity across areas. Non-parametric procedures were used to analyze the empirical distribution for the observed statistics and compare the results in six situations (low/medium/high utilization rates and low/high variability). A small scale simulation study was conducted to assess the capacity of each statistic to discriminate between different scenarios with different degrees of variation.

**Results:**

Bootstrap techniques proved to be good at quantifying the difference between the null hypothesis and the variation observed in each situation, and to construct reliable tests and confidence intervals for each of the variation statistics analyzed. Although the good performance of Systematic Component of Variation (SCV), Empirical Bayes (EB) statistic shows better behaviour under the null hypothesis, it is able to detect variability if present, it is not influenced by the procedure rate and it is best able to discriminate between different degrees of heterogeneity.

**Conclusion:**

The EB statistics seems to be a good alternative to more conventional statistics used in small-area variation analysis in health service research because of its robustness.

## Background

Small Area Variation Analysis (SAVA) is a method used in health services research to describe how rates of healthcare utilization vary across geographic areas [1]. While utilization rates can be calculated to summarize non-binary events (hospital days, costs), they are usually computed to represent counts (procedures, hospital admissions). Studies based on SAVA have documented dramatic variations across areas in the use of medical and surgical procedures, showing that the amount and type of medical care that the individuals of a population receive depend on where they live. The principal finding of these studies remains unchanged: for medical care, geography is destiny [2]. SAVA methods are, thus, used extensively to characterize medical care, assuming that high variability conditions are associated with higher uncertainty and supply-sensitive care [3], and Wennberg constructed an influential general theory describing how to detect physician uncertainty from the variation in small area analysis [4].

The importance of these studies in terms of their impact on policy-making contrasts with the dearth of work testing the validity of the SAVA statistics themselves. Very little has been done to determine whether higher than randomly expected variability across areas is in fact detected, or whether certain procedures are more variable than others [5-12]. Not surprisingly, statistical analysis of area variations in health service research is often informal, consisting of plots and maps illustrating admission or surgery rates by healthcare area, and statistics with important statistical limitations [13,14].

Two groups of statistics of variation are commonly used: those that describe the distribution of rates (based on standardization by direct method) and those that use differences between expected and observed cases (based on indirect standardization). Statistics among the former usually include the high-low ratio or extremal quotient (EQ, maximum rate divided by minimum rate) [5,7], and the unweighted (CV) and weighted (CVw) coefficients of variation.[7] Among the latter, the systematic component of variation (SCV) proposed by McPherson *et al*, [15] and the chi-squared statistic (χ^2^) [7,10] are the most frequently used. Through simulations studies, these statistics have been shown to be sensitive to specific characteristics, such as the prevalence of the procedure or condition, the possibility of multiple admissions, the number of areas considered, and the population size of small areas [7]. Simulation studies have also shown that the expected variation, when the hypothesis of homogeneity in rates is true, can be surprisingly large; especially, in low-incidence procedures or when readmissions are frequent [8]. Therefore, it is important to assess how far variation estimates are from the null hypothesis, and how precise the statistics are in each particular situation.

Several studies conducted by Diehr and her colleagues, including work assessing the power of the tests applied [9], the effect of multiple admissions [10], and the comparison of variability between procedures [11], have contributed extraordinarily to the advance in SAVA methodology. Nevertheless, these authors were "*unable to recommend a single good descriptive for small-area analysis*" [8]. Additionally, SAVA statistics methodology has still limitations. The simulation procedure constructed by Diehr *et al *did not take into account the well-known age and sex variability of most health conditions (although these authors developed an interesting approach in one appendix) [7]. On the other hand, it is important to study not only the behaviour of the statistics under the null hypothesis along with their power, but also to evaluate their capacity to discriminate between procedures with different variability. Finally, Diehr carried out the analyses using a setting with a small number of geographic areas, and where utilization rates were several times higher than the usual rates observed in the Spanish context.

Our work has pursued three objectives: 1) to determine whether variation in rates between areas is higher than would be expected by chance, complementing the study under the null hypothesis of homogeneity by constructing confidence intervals for the observed statistics based on non-parametric bootstrap techniques; 2) to estimate the power of the variation statistics; and 3) to evaluate the ability of different statistics to compare variability among procedures regardless of their rates. Additionally, we extended the simulation procedure to other barely used statistics, such as the empirical Bayes (EB) statistic, that was first proposed in this context by Shwartz *et al*.[12]. The EB focuses on estimating rates rather than on testing significance, and the model underlying this statistic has been applied in some SAVA papers [16,17]. We also considered the Dean (DT) [18], and Bohning statistics (BT) [19], which have been used to test if geographical variation in rates is larger than that assumed under homogeneity in mortality studies [20], but have not been applied yet in health-services research variation analysis.

## Methods

### Database, small geographic areas and procedures under study

We used data from the Atlas of Variations in Medical Practice in the Spanish National Health System (NHS) [21], a research project designed to inform Spanish decision-makers on differences in such parameters as hospital admissions or surgery for specific conditions across geographic areas (see: ). The Spanish Atlas emulates the Dartmouth Atlas of Health Care Project [22]. Hospital Discharge Administrative Databases in 2002 (calendar year), with additional data from ambulatory surgery registries, were used to build the numerator of the rates. These administrative databases, produced by every acute care hospital in the Spanish NHS, provide the following information from every single admission: age, sex, admission and discharge dates, diagnosis and procedure codes [International Classification of Diseases 9^th ^revision Clinical Modification codes (ICD9CM)], and postal codes identifying the patient's area of residence. This latter was used to assign every patient admitted in a hospital to the Healthcare Area in which he lives.

Denominators to calculate population rates came from the Municipal Register of Inhabitants of the Spanish National Institute of Statistics' for 2002. The small geographic areas used corresponded to the "Healthcare Areas" defined by the Health Departments of the 14 Autonomous Regions which participated in the Atlas Project. In total, 147 areas including 75% of the 2002 Spanish population were used. Table 1 shows the population distribution across the Healthcare Areas: 27% of the country's Healthcare areas had less than 100,000 people and only 4% had over a million.

**Table 1:** Population distribution of the geographical areas

Inhabitants	Frequency	Percentage
10,000 – 49,999	9	6.1%
50,000 – 99,999	31	21.1%
100,000 – 149,999	29	19.7%
150,000 – 199,999	13	8.8%
200,000 – 249,999	14	9.5%
250,000 – 299,999	18	12.3%
300,000 – 399,999	17	11.6%
400,000 – 499,999	10	6.8%
500,000 – 999,999	0	0.0%
1,000,000 – 1,500,000	6	4.1%

Total	147	100%

We chose six procedures (pacemakers implant, appendectomy, admission for hip fracture, lower extremity amputation, inguinal hernia repair and knee replacement) taking into account both their utilization rate and their variability. We labelled them as low or high-variation procedures, and as performed at high, medium or low utilization rate. This classification was carried using as reference the whole set of procedures analyzed in the Spanish Atlas project, which add up a total of 35. Hence, by combining the two dimensions, we were able to reproduce six different situations that embrace all of the major cases concerning SAVA studies. ICD9CM codes and inclusion criteria for defining numerators are shown in Table 2.

**Table 2:** Codes of the ICD9MC used for selecting cases.

**Procedure**	**ICD9CM codes**	**Observations**
Appendectomy	47.0x; 47.1x	All appendectomies, including laparoscopic and incidentals.
		
Inguinal hernia repair	53.0x; 53.1x; 53.2x; 53.3x	Uni or bilateral repair, with or without mesh, of femoral or inguinal hernias.
		
Lower extremity amputation	84.10 to 84.17	Lower extremity amputation at any level.
		
Hip fracture	820.xx	Only emergency admissions.
		
Knee replacement	81.54; 81.55	Total or partial knee replacement
		
Pacemaker implant	37.80; 37.81; 37.82; 37.83	Pacemaker implant, permanent or not, in programmed or emergency admissions.

ICD9CM: International Classification of Diseases 9 revision Clinical Modification; The "x" indicates all the range of digits after the corresponding code.

### Analysis

Extremal Quotient [7], Coefficient of Variation [7], Weighted Coefficient of Variation [11], Systematic Component of Variation [15], Empirical Bayes variance component [12], χ^2 ^statistic [11], Dean statistic [18], and Bohning statistic [19] were all studied. Because some of the Spanish Atlas' calculations exclude the 5% of extreme standardized rates for each tie [21], we have also eliminated the outliers beyond of the 5–95 percentiles, and labelled our statistics as EQ_5–95_, CV_5–95_; CVW_5–95 _and SCV_5–95_. The formulation of the statistics is given in Additional file 1. The EQ, CV and CVw use direct age-standardized rates for each *i-th *Healthcare Area, denoted by *DSR*_*i *_for *i *= 1, ..., *I*, and all three are well-known measures of variation in general contexts. The remaining statistics use the observed and expected cases per area, denoted by *y*_*i *_and *e*_*i *_respectively. These expected cases were derived based on the age-specific rate for 8 groups (0–24, 25–44, 45–64, 65–69, 70–74, 75–79, 80–84, 85 years and over) and the sex stratum in the standard population, which was the population from the 147 healthcare areas under study. More precisely, *e*_*i *_= ∑_*j*, *k*_*n*_*ijk *_*R*_*jk*_, where n_ijk _is the population in area i, age group j and sex stratum k, and R_jk _is the age-sex specific rate for the whole region under study. Hence, the quotient of the observed to the expected number of cases is the indirect Standardized Utilization Ratio, SUR_i _= y_i_/e_i _for the *i-th *Healthcare Area. This quotient is in fact the maximum-likelihood estimator of r_i_, the unknown relative risk of suffering a given surgical procedure in the area, under the assumption that y_i _~Poisson(e_i_r_i_) independently for each *i-th *Health Area. The Poisson distribution is frequently adopted because the Bernoulli process at the individual level (surgery vs non surgery) becomes a Binomial process at the area level, which can be approximated by the Poisson distribution when rare events are modelled [10]. Hence, the null hypothesis indicating an homogenous risk surface for the whole region can be represented by the model y_i_~Poisson(e_i_r), with *r *the common risk. The X^2^, DT and BT versions applied here were derived to detect heterogeneity with respect to this homogeneous Poisson model. Finally, the SCV and the EB statistics are derived under a more general framework where the number of admissions per area is modelled hierarchically in a two-step procedure. The first step assumes that, conditional on the risk r_i_, the number of counts y_i _follows a Poisson distribution, y_i_|r_i _~Poisson(e_i_r_i_), whereas in the second one, heterogeneity in rates is modelled adopting a common distribution *π *for the risk r_i _(or for its logarithm), *r*_*i*_~ *π(r|****θ****)*, with ***θ ***the vector of parameters of the density function. Whereas the derivation of the SCV does not require a parametric form for *π*, as the SCV is precisely the moment estimator of the variance in the distribution of *π *[15], the EB statistics is based on the assumption that the log-relative risks are normally and identically distributed, log(r_i_) ~N(μ, σ^2^). This last model, called multivariate Poisson log-normal model or exchangeable model, is widely used in the disease mapping literature [23,24], and can be easily extended to accommodate spatial autocorrelation [25,26]. The marginal distribution of this model is not available in closed form, and two approaches can be used to derive estimates for the parameters such as the variance component σ^2 ^and predictions for the random effects representing r_i_. These are the Empirical Bayes (EB) approach, which can be accomplish using the Penalized Quasi Likelihood method [27], or the Full bayes (FB) approach [25,26] for which prior distributions for the parameters are required. The EB statistics used in this paper is the estimate of the variance component σ^2 ^derived under the EB approach, but similar results would have been obtained under the FB approach. [28]. Under the null hypothesis of homogeneity among rates, both the SCV and the EB statistics would be zero.

#### Assessing the null hypothesis of homogeneity via bootstrap sampling methods

The null hypothesis of homogeneity tested here is that the expected admission rate for each procedure is the same in all counties, so that differences in observed rates are no bigger than that expected by chance, assuming an underlying Poisson process to model admission counts [10]. This hypothesis has been tested using bootstraping [29,30], which is a resampling procedure that estimates the properties of an estimator (such as its variance) by sampling from an approximating empirical distribution. There are two types of bootstrap procedures, for parametric and non-parametric inference. The former can be adopted when exists a parametric model from which samples can be randomly generated to derive the empirical distribution of the statistic, whereas the latter relies on the discrete empirical distribution obtained by random sampling with replacement from the original dataset. Given that the homogeneous Poisson model (or alternatively the normal model with common rate) was assumed under the null hypothesis, the parametric bootstrap procedure was used to derive upper and lower limit values from "*R*" random samples generated from the hypothesis being tested. Even though the same philosophy was first proposed by Diehr *et al*. [9], we implemented as additional analysis the age-sex adjustment. The source of information used for each statistics (i.e., standardized rates vs. observed-expected cases) was also considered in the analysis. The steps for carrying this analysis out are shown in Additional file 2.

#### Deriving confidence intervals for statistics of variation via nonparametric sampling methods

In order to assess the alternative hypothesis, confidence intervals for the observed statistics were derived. Here we used non-parametric methodology in order to avoid parametric assumptions about the distribution of both rates and observed cases. Thus, sampling with re-sampling R times from the observed standardized rates sample or from the observed-expected cases paired sample (depending on the statistic) was used to calculate statistics for each of the R simulated samples. This made it possible to obtain confidence intervals from the percentile 2.5 and 97.5 as before.

#### Assessing power and ability to discriminate between procedures with different variability by means of a small scale simulation study

In order to derive the ability of the aforementioned statistics to distinguish different degrees of variability, we simulated several situations that emulate different types of induced variability. This exercise pursued three objectives: First, to assess the statistical power of each one of the above described statistics, which in this case represents the probability of detecting geographic variability when it is present. Second, to evaluate whether any were better than the others at distinguishing and ordering the six different scenarios with regard to the degree of variability; and finally, to study how different rates influenced statistics of variation when they are used to compare procedures according to their variability.

Apart from the scenario named H_0 _representing the homogeneous Poisson model with a common risk surface y_j _~Poisson(e_j_r_j_), with r_j _= 1 for all j in 1, ..., J, and generated as described in Additional file 2, six additional scenarios with different degrees of variability were designed. The population structure was based on that observed in the real geographical pattern with I = 147 areas, whereas the expected counts were derived using the overall age-sex specific rates for the most frequent (hip fracture) and the least frequent (lower extremity amputation) procedure. While most of the regions were assumed to have homogeneous rates, an artificially elevated risk was induced in a randomly selected group of areas. These was carried out using two sources of additional variation: incrementing the risks of the selected areas to r_j _= 1.2 (S_1_–S_3_) or r_j _= 1.6 (S_4_–S_6_) (and equivalently their mean rates in 1.2p and 1.6p respectively), and varying the number of these areas with induced elevated risk, being 10 (S_1 _and S_4_), 20 (S_2 _and S_5_), and 40 Healthcare Areas (S_3 _and S_6_) out of the 147. Counts in all background areas (all but these 10, 20 or 40 respectively) were generated from the null model with a common underlying rate aforementioned. Hence, the scenarios were numbered from the lowest to the highest expected variability S_1_<S_2_<S_3_<S_4_<S_5_<S_6_.

Once the scenarios were designed, 2000 samples were simulated from the null distribution following the procedure previously described and named H_0_. The critical value of the tests was estimated using the 95-th percentile of the empirical distribution of the statistics, whereas confidence interval limits were obtained form the same distribution using percentiles 2.5 and 97.5. Another 2000 samples were simulated from each scenario S_1_–S_6_, and the empirical distribution of the statistics was derived. This allowed us to obtain not only the empirical statistical power of the test for each scenario, by calculating the proportion of values greater than the critical values obtained in H_0 _(the proportion of times that the null hypothesis is surpassed in each scenario), but also the confidence intervals for the statistics in each scenario using percentiles 2.5 and 97.5 of the empirical distribution.

## Results

### Real case study results

Table 3 shows the rates and the observed statistics of variation for the six procedures under study. Rates varied from 3.77 pacemaker implants to 10.57 hip fracture admissions per 10,000 inhabitants in procedures presumed to show low variation, and from 2.33 lower extremity amputations to 7.39 knee replacements per 10,000 inhabitants, in procedures presumed to have high variation. We could not calculate the EQ for some of the procedures because some of the Healthcare Areas had 0 cases. For this reason we excluded the EQ (not the EQ_5–95_) from the simulation procedures. The exclusion of 5% of outlying areas on each side of the distribution notoriously reduced the value of practically all the statistics, including the SCV. This occurred in procedures with low and high variation, not depending on prevalence rates. Some statistics, such as the χ^2^, and the Dean and Bohning tests, tended to have higher values as the overall rate increases, regardless of the underlying variability.

**Table 3:** Number of cases, rates by 10,000 inhabitants and observed statistics of variation in procedures of low and high variability

**A: Low variability**						
	
	**Pacemaker**		**Appendectomy**		**Hip Fracture**	
	
	Estimate	CI	Estimate	CI	Estimate	CI
∑*y*_*i*_	11973		28164		33851	

**Rate**	3.77		8.90		10.57	

**EQ**	NC	--	20.61	3.20; 20.61	4.11	3.28; 4.11

**EQ_5–95_**	3.15	2.49; 3.58	2.49	2.20; 2.74	2.37	2.00; 2.69

**CV**	0.38	0.33; 0.43	0.30	0.25; 0.34	0.26	0.24; 0.29

**CV_5–95_**	0.27	0.24; 0.31	0.22	0.19; 0.25	0.21	0.19; 0.24

**CVw**	0.33	0.28; 0.38	0.30	0.25; 0.34	0.27	0.23; 0.31

**CVw_5–95_**	0.26	0.23; 0.31	0.24	0.19; 0.26	0.21	0.19; 0.24

**SCV**	0.12	0.08; 0.16	0.11	0.08; 0.16	0.07	0.06; 0.08

**SCV_5–95_**	0.05	0.04; 0.08	0.06	0.04; 0.08	0.04	0.03; 0.06

**EB**	0.13	0.08; 0.20	0.08	0.06; 0.10	0.07	0.05; 0.09

**χ^2^**	1306.85	964.45;1711,39	2330.55	1710.91;3098.22	2394.66	1820.36;3017.15

**Bohning**	68.46	47.88; 92.28	136.98	93.89; 187.56	131.32	96.41; 167.70

**Dean**	67.44	40.11; 107.10	164.03	97.40; 246.83	132.92	80.47; 203.67

**B: Low variability**						
	
	**Lower Ext. Amput**.		**Hernia Repair**		**Knee replacement**	
	
	Estimate	CI	Estimate	CI	Estimate	CI

∑*y*_*i*_	7022		21101		23257	

**Rate**	2.23		6.67		7.39	

**EQ**	25.59	7.04; 25.59	NC	--	29.13	12.09; 29.14

**EQ_5–95_**	4.11	3.54; 5.10	4.06	3.28; 4.90	5.61	4.39; 8.66

**CV**	0.42	0.36; 0.47	0.41	0.37; 0.45	0.49	0.44; 0.55

**CV_5–95_**	0.33	0.27; 0.37	0.34	0.29; 0.37	0.39	0.33; 0.44

**CVw**	0.40	0.34; 0.46	0.41	0.35; 0.47	0.48	0.41; 0.54

**CVw_5–95_**	0.31	0.27; 0.38	0.34	0.29; 0.38	0.39	0.32; 0.45

**SCV**	0.20	0.14; 0.28	0.17	0.13; 0.21	0.25	0.18; 0.32

**SCV_5–95_**	0.13	0.07; 0.20	0.11	0.08; 0.13	0.14	0.10; 0.19

**EB**	0.17	0.12; 0.24	0.17	0.13; 0.22	0.27	0.19; 0.35

**χ^2^**	1171.47	850.08;1534.95	3531.00	2723.57;4438.64	5164.25	3766.17;6898.34

**Bohning**	59.35	41.85; 78.83	195.84	146.27; 247.92	289.92	207.33; 384.14

**Dean**	54.57	32.13; 89.46	222.09	149.79; 314.23	275.13	170.29; 424.52

CI: Confidence interval; EQ: extremal quotient; CV: Coefficient of variation; CVw: weighted coefficient of variation; SCV: Systematic Component of Variance; EB: Empirical Bayes; Statistic with the subindex 5–95 have been estimated excluding the 5% of areas with rates under percentile 5 and over percentile 95 for each procedure; NC: Not Calculable.

**Figure 1:**
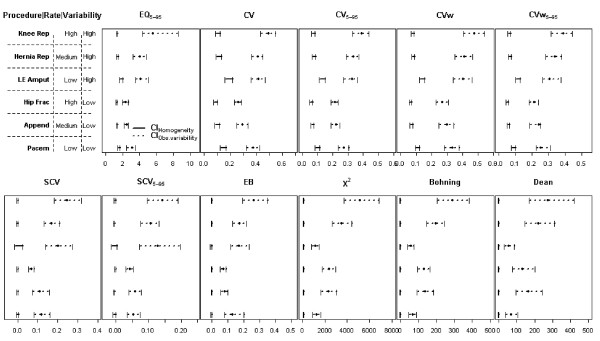
**Point estimates and confidence intervals for the statistics of variation applied to the six medical procedures under the null hypothesis (homogeneity across Healthcare Areas, continuous line) and the alternative hypothesis (observed rates, dotted line)**. Procedures are sorted from high to low rate and grouped by low and high variability, see left axis. CI_Homogeneity_: estimates and confidence intervals when the null hypothesis of homogeneity holds (continuous line); CI_Obs.variability_; estimates and non-parametric confidence intervals for the statistics when the observed variability is considered (dotted line); EQ: extremal quotient; CV: Coefficient of variation; CVw: weighted coefficient of variation; SCV: Systematic Component of Variance; EB: Empirical Bayes. Statistic with the subindex 5–95 have been estimated excluding the areas with rates under percentile 5 and over percentile 95 for each procedure.

Figure 1 presents the point estimates for each procedure, together with the parametric confidence intervals when the null hypothesis of homogeneity holds (continuous line), and the non-parametric confidence intervals for the observed statistic (dotted line). This figure shows that under the assumed null hypothesis, behaviour differs depending on whether the statistics of variation are based on rates (upper row) or on observed-expected cases (lower row). In particular, the former present wider confidence intervals for the procedures with the lowest rates (lower extremity amputation and pacemaker implant); furthermore, they are "shifted to the right" for these procedures. In contrast, for those statistics based on the observed-expected cases, no apparent differences related with the underlying rate are found, with the exception of the SCV, with a notably wider interval for the less frequent procedure. In these cases, the χ^2^, the Dean and the Bohning statistics show narrow confidence intervals.

Regarding the observed variation, confidence intervals for the observed statistics are wider than their null counterparts, and these discrepancies in amplitude are higher in the statistics based on the observed-expected comparisons than in the rate-based statistics. Of note is the agreement among the statistics in detecting which is the most variable procedure, all suggesting that knee replacement has the highest point and the widest confidence interval estimates, being very far removed from the null hypothesis. However, this agreement is not observed when trying to elucidate which procedure presents the lowest variability. While most statistics detect that admissions for hip fracture and appendectomy seem to have the lowest point estimates, the χ^2^, Bohning and Dean tests suggest that pacemaker implant or lower extremity amputation, the two procedures with the lowest rates, appear to have lower point estimates than those obtained for the rest of the procedures. Representing together confidence intervals of the statistics and those obtained under homogeneity in the same graph allows us to derive more reliable conclusions regarding the underlying variability. Specifically, the closer they are, the less probability for systematic variation (i.e., beyond chance). Note also that excluding the 5% of extreme rates in some statistics seems negligible with regard to the comparison between null and observed intervals, because the expected variability depicted is lower when excluding them both under the null hypothesis and under the observed variability.

### Small scale simulation study results

**Figure 2:**
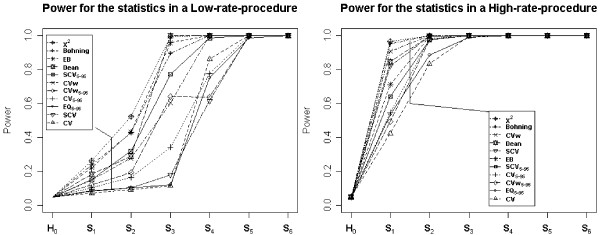
**Power for the statistics for each scenarios**. EQ: extremal quotient; CV: Coefficient of variation; CVw: weighted coefficient of variation; SCV: Systematic Component of Variance; EB: Empirical Bayes. Statistics with the subindex 5–95 have been estimated excluding the areas with rates under percentile 5 and over percentile 95 for each procedure.

The empirical power of the statistics is presented in Figure 2 for a high-rate (hip fracture) and a low-rate (lower extremity amputation) procedure. In both cases, the most powerful were those based on the observed-expected relationship, such as χ^2^, Bohning, EB and Dean statistics. However, the statistics' behaviour changed radically depending on the rate of the procedure, and were more powerful when a high-rate procedure was considered.

**Figure 3:**
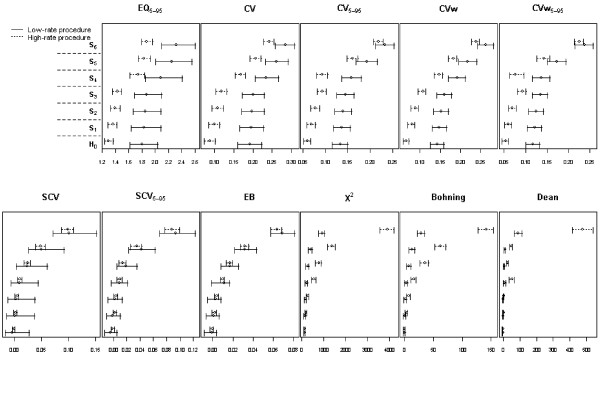
**Point estimates and confidence intervals for the statistics using six scenarios of variation and two different rates**. EQ: extremal quotient; CV: Coefficient of variation; CVw: weighted coefficient of variation; SCV: Systematic Component of Variance; EB: Empirical Bayes. Statistic with the subindex 5–95 have been estimated excluding the 5% of areas with rates under percentile 5 and over percentile 95 for each procedure.

Confidence intervals for the statistics under the six scenarios and for the high and low rate procedures are given in Figure 3. With regard to their capacity to distinguish between alternative scenarios, most statistics have a parabolic shape from H_0 _to S_6_. They are, thus, able to distinguish between the alternatives, and the higher the variability induced by raising the rates of the non-homogeneous risk regions (scenarios S_4_–S_6 _relative to scenarios S_1_–S_3_) or by increasing the number of regions with non-homogeneous risk (S_3 _and S_6 _compared to the rest), the easier it is to detect differences between scenarios. The EQ_5–95 _statistic seems to be the least able to distinguish between different degrees of variability, whereas the CVw, EB, χ^2 ^and the Bohning test are the best for this purpose when the rate of the procedure is high (dotted line). These tests do not perform as well when this rate is low in low-variability scenarios S_1_–S_3_. Note also that there are not remarkable differences between the performance of the statistics based on rates and their counterpart statistics after excluding the 5% of extreme rates of each tie, apart from the reduction on the punctual estimates and the slight reduction on the amplitude in the later, more apparent in the low-rate procedure setting.

Figure 3 also reveals some interesting findings with regard to the differences between the statistics' behaviour when the procedure rate is considered. To compare the variability of different procedures, statistics must not be affected by the procedure rate. As we can see in Figure 3, only the SCV, SCV_5–95 _and EB statistics uphold this assumption. Furthermore, EB has the narrowest confidence intervals, particularly for the low-rate procedures compared to the SCV and SCV_5–95_. This finding explains why EB is more powerful than the others when low rate procedures are studied. In contrast, all the statistics based on rates (upper row) show that a high-rate procedure would always be considered to have lower variability than a low-rate procedure. For the χ^2 ^and the Bohning and Dean statistics the opposite is true, and only under H_0 _they are equivalent.

All analyses were carried out using the free statistical package R 2.4.0. [31]

## Discussion

Our first objective was to analyze whether variation among areas is higher than would be expected by chance. Our findings are not completely consistent with previous literature, in which the expected variability when the null hypothesis of homogeneity is true was said to be surprisingly large [7,8,10]. In our work, practically all the statistics under the null hypothesis have narrow intervals which are close to the zero value (or 1 for the EQ_5–95_) compared to those derived from the observed data, which are shifted to the right (see Figure 1). The distance between the upper limits of the null intervals null and the lower limit of the observed intervals is present in all procedures and occurs for all statistics, indicating that we observed more variability than the expected by chance even for some procedures that are known to have low variation, such as hip fracture. This discrepancy with previous studies could be related to the size of our sample (n = 147 Healthcare Areas), which was larger than the sample size used in the reference article by Diehr *et al *(n = 39 counties) [7], and suggests that significant variation is expected to be found for most procedures in studies with large number Healthcare Areas, such as the Dartmouth Atlas of Health Care, with more than 300 hospital reference areas, or the Spanish NHS Atlas of Variations, with more than 140 areas, making the interpretation of procedure variations difficult when the significance of statistics such as the X^2 ^is given. Furthermore, the fact that some statistics perform differently under the null hypothesis depending on the rate of the procedure (see Figure 1) indicates that it is not adequate to provide only the observed statistics, because the same observed value may represent different degrees of variability depending on the procedure rate. These are relevant aspects that suggest that providing both the null and observed performance jointly over a simple observed descriptive statistic value or a simple p-value is more appropriate.

Another interesting question broached by Diehr [7] and other authors [32] was the better performance of the χ^2 ^statistic (compared to others) because of its lower dependence on population size, condition rates or readmissions. In fact, Diehr *et al *recommended its use. However, these findings were only described under the null hypothesis. Our work has confirmed the aforementioned behaviour of the χ^2 ^statistic (see scenario H_0 _on Figure 3), but its stability for different rates diminished when the alternative was true (see S_1 _to S_6 _scenarios, Figure 3). In fact, the χ^2 ^statistic appears to have higher values as a procedure rate increased, regardless of the actual underlying variability.

With regard to the new statistics we have tested, Dean and Bohning tests performed almost identically to χ^2^, as they all were designed to detect departures from homogeneity rather than to discriminate among degrees of variability. In fact, the expected value of the first statistic under the null hypothesis is the number of area minus one, whereas the other two have asymptotically a standard normal distribution; all the three show a good performance under the null hypothesis, but are highly dependent on the procedure rate under alternative scenarios. On the other hand, the results obtained with EB were closer to those find using SCV and SCV_5–95_. This concordance among statistics was also expected since the first three tests are based on the discrepancy between observed and expected cases given the homogeneous Poisson model, while the other three are based on a generalized linear mixed model where the area-specific effect is the random effect. Overall, the last three statistics, and especially the EB, show a good performance both under the null and the alternative hypotheses, being stable even when procedure rates change. EB's good behaviour is consistent with Shwartz's results [12], and confirms that this statistic should become an essential part of SAVA studies.

The second aim of our paper consisted on estimating the statistical power of the variation statistics, and our results are consistent with Diehr's [9]. There were relevant differences between theirs and our scenarios of study: their procedures were more prevalent than ours (18 per 10,000, while ours ranged from 2.2 to 10.6 per 10,000 depending on conditions) and their sample size was smaller (39 counties compared to our 147 Healthcare Areas). In spite of this, the outcomes of both studies point in the same direction: the χ^2 ^test appears to have the most statistical power, and the CV and EQ the least. Nevertheless, Diehr's work did not evaluate other statistics such as the EB, which has practically the same power that the widely recommended χ^2 ^and performs better in terms of stability under the alternative hypothesis.

With regard to our third objective, our work sought to compare variation profiles between different procedures. Traditionally, this objective in SAVA studies is pursued by using simple dot plots, descriptive statistics without significance testing or ratios between the SCV of the revised procedures and the SCV of hospitalization for hip fracture, a known low-variation condition [22,33,34]. In our work, all the statistics evaluated seem to agree when the procedure or condition presents high variability. This finding is important because it confirms that conditions identified as highly variable remain consistent across statistics, suggesting that SAVA analysis is a useful method for targeting conditions for intervention or further study. Moreover, it is important to be aware that the sensitivity to low-rate procedure of the χ^2 ^statistic (and the Dean and Bohning tests) may suggest low variability, as seems to have happened in the case of lower extremity amputation. Because of this problem, the χ^2 ^statistic appears not to be the best choice in SAVA studies.

In order to truly compare variation among procedures, SAVA studies must use reliable statistics that are able to detect variability when it exists. These statistics must perform robustly when there are differences in utilization rates among the procedures, and when small-sized samples are studied. The main conclusion of our study is that the SCV and, mainly, the EB statistic have been shown to be the best, because they do not seem to be influenced by the utilization rates of the conditions or procedures under study (a relevant advantage when conditions of very different rates are compared), and because it is able to accurately discriminate between different degrees of heterogeneity (see confidence intervals in S_1 _to S_6_, Figure 3).

Our work has not included all the statistics suggested in the literature, but has concentrated on those most widely used, and those that are commonly used in other contexts, such as mortality analysis. Diehr *et al *proposed the use of the CVA [7], which was recommended when procedures had high prevalence rates. They showed that the CVA, which is derived from an analysis of variance where the response variable is the number of admissions for each person in each area and the area is the random effect, do not correlate with the procedure rate in contrast to other estimates of variation (CV, CVw). Our study corroborates the influence of prevalence in the latter statistics and also shows that neither the SCV nor the EB have this limitation. Furthermore, the underlying Poisson distribution assumed for SCV and EB statistics [12] was considered more appropriate than normal assumptions with equal variances needed for the CVA calculations. In particular, the peculiarities of the model underlying the EB computation, that takes into account the reliability of each area to weight the information each of them gives to the pooled variation, encouraged us to prefer the properties of the EB to be used in these studies. Smoothing techniques such as the EB are now dominating the literature in disease mapping, and can be easily programmed using standard software such as R.

Our work has several limitations. First, we have not addressed the analysis considering recurrent events (i.e. readmissions). Although the six procedures under study are not likely to have recurrent events in a one-year period (with the exception of lower extremity amputation) it is important to note that the possibility of multiple counts in recurrent events violates the assumption of independence of Poisson events. The variance may be higher and the standard approaches may not account for the extra variation, underestimating variability [7,10,35]. Different approaches to overcome this problem have been proposed in the literature. These include the Multiple Admission Factor [10], or the use of other distributions rather than Poisson. Additionally, the assumed null model does not consider the variability that may be present due to disease prevalence variation. This could have been incorporated with models accounting for overdispersion and estimated if reliable outpatient registers had been available. Although some interesting attempts are being carried out in this direction [16,36], at present these registers are not reliable enough in our setting. The approach presented here has neither taken into account the spatial autocorrelation that may exist in the data, because a comparison of smoothing techniques incorporating it did not suggest that its inclusion would lead to different results, given the high populated regions usually considered in health service research studies. Nevertheless, the EB estimate can easily be extended to account for spatial correlation [20] and it provides estimates close to the full-Bayes counterparts [28,37], so that we recommend SAVA studies to go in this direction to be of benefit for the advances produced in disease mapping studies. Another limitation is related with the simulation study, where only two variation sources were used, the number of heterogeneous areas above the overall level (10, 20 or 40) and the magnitude of differences (RR = 1.6 or RR = 1.2), and two different procedure rates were considered. It may happen that other settings with different number of regions, different rates, different population distributions or different degrees of induced variability could have led to different results.

Despite the importance of our findings, some questions remained unsolved. With the exception of the EQ, the remaining statistics assessed in this work do not provide information easily translated into action. Unfortunately, while the EQ appears to be the most intuitive statistic, it is also the worst one in terms of sensitivity and robustness. It is, further, also difficult to build when considering areas with no cases. As Coory and Gibberd note [38], we need new measures for reporting the magnitude and impact of small-area variation in rates. In the meantime, it is worth drawing health services researchers' attention to the importance of using adequate measures of its estimation.

## Conclusion

For this reason, and in conclusion, we recommend: 1) to use bootstrap techniques to obtain a joint picture of the observed variability and that obtained under homogeneity, as they provide a complete and reliable measure of the magnitude of variation; 2) to be careful with the interpretation of some statistic estimates, particularly for the rate-based statistics, as their performance differ even under homogeneity depending on the procedure rate: and 3) when variability of different procedures needs to be compared, SCV and specially, EB statistic, are the most robust measures, overcoming problems derived from differences in procedures prevalence rates.

## Competing interests

The authors declare that they have no competing interests.

## Authors' contributions

BI, EBD and SP are guarantors of the study, had full access to all the data, and take responsibility for the integrity and the accuracy of the analysis and results. BI, JL, EBD, SP and FAB contributed to the conception and the design of the article. NML acted as data-manager of the study. BI, JL and BGLV contributed to the study analysis. BI, SP, JL and EBD interpreted the results, and drafted the article. All the authors read and approved the final manuscript.

## Pre-publication history

The pre-publication history for this paper can be accessed here:



## Supplementary Material

Additional File 1 **Table s1**. Formulation of the descriptive statistic. Click here for file

Additional File 2 **Table s2**. Schematic Diagram of Simulation under the null hypothesis of homogeneity*. Click here for file

## References

[B1] Diehr P, Armitage P, Colton T (2005). Small Area Variation Analysis. Encyclopedia of Biostatistics.

[B2] Wennberg DE (1998). Variation in the delivery of health care: the stakes are high. Ann Intern Med.

[B3] Fisher ES, Wennberg JE (2003). Health care quality, geographic variations, and the challenge of supply-sensitive care. Perspect Biol Med.

[B4] Wennberg JE, Sechrest L, Perrin E, Binker J (1990). Small area analysis and the medical care outcome problem.

[B5] Kazandjian VA, Durance PW, Schork MA (1989). The extremal quotient in small-area variation analysis. Health Serv Res.

[B6] Diehr P, Sechrest L, Perrin E, Binker J (1990). Small area analysis: the medical care outcome problem.

[B7] Diehr P, Cain K, Connell F, Volinn E (1990). What is too much variation? The null hypothesis in small-area analysis. Health Serv Res.

[B8] Diehr P, Grembowski D (1990). A small area simulation approach to determining excess variation in dental procedure rates. Am J Public Health.

[B9] Diehr P, Cain KC, Kreuter W, Rosenkranz S (1992). Can small-area analysis detect variation in surgery rates? The power of small-area variation analysis. Med Care.

[B10] Cain KC, Diehr P (1992). Testing the null hypothesis in small area analysis. Health Serv Res.

[B11] Diehr P, Cain K, Ye Z, Abdul-Salam F (1993). Small area variation analysis. Methods for comparing several diagnosis-related groups. Med Care.

[B12] Shwartz M, Ash AS, Anderson J, Iezzoni LI, Payne SM, Restuccia JD (1994). Small area variations in hospitalization rates: how much you see depends on how you look. Med Care.

[B13] Diehr P (1984). Small area statistics: large statistical problems. Am J Public Health.

[B14] Julious SA, Nicholl J, George S (2001). Why do we continue to use standardized mortality ratios for small area comparisons?. J Public Health Med.

[B15] McPherson K, Wennberg JE, Hovind OB, Clifford P (1982). Small-area variations in the use of common surgical procedures: an international comparison of New England, England, and Norway. N Engl J Med.

[B16] Shwartz M, Peköz EA, Ash AS, Posner MA, Restuccia JD, Iezzoni LI (2005). Do variations in disease prevalence limit the usefulness of population-based hospitalization rates for studying variations in hospital admissions?. Med Care.

[B17] Havranek EP, Wolfe P, Masoudi FA, Rathore SS, Krumholz HM, Ordin DL (2004). Provider and hospital characteristics associated with geographic variation in the evaluation and management of elderly patients with heart failure. Arch Intern Med.

[B18] Dean CB (1992). Testing for overdispersion in Poisson and binomial regression models. J Am Stat Assoc.

[B19] Böhning D (2000). Computer-assisted analysis of mixtures and applications: Meta-analysis, disease mapping, and others.

[B20] Ugarte MD, Ibáñez B, Militino AF (2006). Modelling risks in disease mapping. Statistical Methods in Medical Research.

[B21] Librero J, Rivas F, Peiró S, Allepuz A, Montes Y, Bernal-Delgado E (2005). Metodología en el Atlas VPM. Atlas Var Pract Med Sist Nac Salud.

[B22] Wennberg JE, Cooper MM (1996). Dartmouth Atlas of Health Care in the United States.

[B23] Lawson AB, Biggeri AB, Bohning D, Lesaffre E, VIel JF, Clark A, Schlattmann P, Divino F (2000). Disease mapping models: an empirical evaluation. Statistics in Medicine.

[B24] Wakefield J (2007). Disease mapping and spatial regression with count data. Biostastistcs.

[B25] Besag J, York J, Mollié A (1991). Bayesian image restoration with two applications in spatial statistics. Annals of the Institute of Statistical Mathematics.

[B26] Richardson S, Thomson A, Best N, Elliot P (2004). Interpreting Posterior Relative Risk Estimates in Disease-Mapping Studies. Environmental Health Perspectives.

[B27] Breslow NE, Clayton DG (1993). Approximate inference in general linear mixed models. J Am Stat Assoc.

[B28] MacNab YC, Farrell PJ, Gustafson P, Wen S (2004). Estimation in Bayesian Disease Mapping. Biometrics.

[B29] Efron B, Tibshirani RJ (1993). An Introduction to the Bootstrap.

[B30] Davison AC, Hinkley DV (1997). Bootstrap Methods and Their Application.

[B31] R Development Core Team (2007). R: A language and environment for statistical computing.

[B32] Carriere KC, Roos LL (1997). A method of comparison for standardized rates of low-incidence events. Med Care.

[B33] Birkmeyer JD, Sharp SM, Finlayson SR, Fisher ES, Wennberg JE (1998). Variation profiles of common surgical procedures. Surgery.

[B34] Wennberg JE, Cooper MM (1999). The Dartmouth Atlas of Health Care in the United States 1999.

[B35] Carriere KC, Roos LL (1994). Comparing standardized rates of events. Am J Epidemiol.

[B36] Peköz EA, Shwartz M, Iezzoni LI, Ash AS, Posner MA, Restuccia JD (2003). Comparing the importance of disease rate versus practice style variations in explaining differences in small area hospitalization rates for two respiratory conditions. Stat Med.

[B37] MacNab YC, Kmetic A, Gustafson P, Sheps S (2006). An innovative application of Bayesian disease mapping methods to patient safety research: A Canadian adverse medical event study. Stat Med.

[B38] Carriere KC, Roos LL (1994). Comparing standardized rates of events. Am J Epidemiol.

[B39] Coory M, Gibberd R (1998). New measures for reporting the magnitude of small-area variation in rates. Stat Med.

