# Supplementary material for: Is there much variation in variation? Revisiting statistics of small area variation in health services research
Source: BMC Health Serv Res. 2009 Apr 2;9:60. doi: 10.1186/1472-6963-9-60 (PMC2676262; doi:10.1186/1472-6963-9-60)
Supplement: Additional File 1 — Table s1. Formulation of the descriptive statistic. [file 1472-6963-9-60-S1.doc]

**Additional file 1. Formulation of the descriptive statistics**

| Extremal quotient (EQ) |  |
| --- | --- |
| Coefficient of Variation (CV) |  |
| Weighted Coefficient of Variation (CVw) |  |
| Chi square | $\mathbf{X}^{\mathbf{2}}\text{ }=\text{ }\left(\sum\frac{(y_{i}-e_{i})^{2}}{e_{i}}\right)\approx X^{2}_{I-1}$ |
| Systematic Component of Variance (SCV) | $SCV=\frac{1}{I}\left(\sum\frac{(y_{i}-e_{i})^{2}}{e_{i}^{2}}-\sum\frac{1}{e_{i}}\right)$ |
| Empirical Bayes (EB) | $\begin{matrix} EB\mathbf{:}\text{Empirical}-\text{Bayes} \text{estimate} \text{of} \sigma^{2} \\ \text{assuming} \log r_{i}\approx N(0,\sigma^{2}),\text{ being }r_{i}\text{ the relative risk} \end{matrix}$ |
| Bohning test (BT) | $Bohning test\mathbf{ }=\text{ }\frac{\frac{1}{I-1}\sum(y_{i}-\overline{SUR} e_{i})^{2}/e_{i}-\overline{SUR}}{\sqrt{2\overline{SUR}\text{/(I-1)}}},with \overline{SUR }=\frac{\sum SUR_{i}}{I}$ |
| Dean test (DT) | $Dean test\mathbf{ }=\text{ }\frac{(y_{i}-e_{i})^{2}-y_{i}}{\sqrt{2\sum e_{i}^{2}}}\approx Z$ |
| DSR: standardized rate; SUR: Standardized utilization ratio=y_i_/e_i_ | |
