# Supplementary material for: Is there much variation in variation? Revisiting statistics of small area variation in health services research
Source: BMC Health Serv Res. 2009 Apr 2;9:60. doi: 10.1186/1472-6963-9-60 (PMC2676262; doi:10.1186/1472-6963-9-60)
Supplement: Additional File 2 — Table s2. Schematic Diagram of Simulation under the null hypothesis of homogeneity*. [file 1472-6963-9-60-S2.doc]

Additional file 2: Schematic Diagram of Simulation under the null hypothesis of homogeneity*

| Step 1 | Calculate statistics given in Additional file 1 from the observed data, T_obs_, for T= EQ, CV, CVw, SCV, EB, χ^2^, BT, DT | |
| --- | --- | --- |
| Step 2 | Simulate R (=2000) bootstrap samples of size the number of areas *I* under H_0_ , which depend on the type of statistic | |
|  | Statistics based on rates | H_0_: $p_{i}^{*}\sim N(p=\sum_{j=1}^{J} p_{j}\pi_{j},\sum_{j=1}^{J} p_{j}(1-p_{j})\pi_{j}^{2}/n_{ij})$; p_i_^*^area-specific proportions, i=1,…, I;  p_j_= standard age-sex rate; *π_j=_* proportion in *j-th* age-sex stratum,*n_ij=_* people in age-sex *j* area i |
|  | Statistics based on cases | H_0_: *y_i_* ~ Poisson(*e_i_*) |
| Step 3 | Compute statistics in Step 1 for each R sample, T_1,…._, T_R_, and derive the empirical distribution | |
| Step 4 | Derive p-values for each statistic: $P_{boot}=\frac{1+\#\{T_{r}\geq T_{obs}\}}{R+1}$; where $\#\{T_{r}\geq T_{obs}\}$={number of simulated T_r_ ≥T_obs_};  Derive confidence intervals for each statistic: IC_100(1-α)%_=(T_(r)100α/2_, T_(r)100(1-α/2)_), with T_(r)i_ the *i*-th quantile of the distribution | |

*The number of bootstrap samples needed to obtain reliable results was assumed to be R=2000.
